# A Modular Neural Network Scheme Applied to Fault Diagnosis in Electric Power Systems

**DOI:** 10.1155/2014/176463

**Published:** 2014-09-17

**Authors:** Agustín Flores, Eduardo Quiles, Emilio García, Francisco Morant, Antonio Correcher

**Affiliations:** ^1^Área de Control Peninsular, CFE, Instituto Tecnológico de Mérida, Departamento de Eléctrica, C10 No. 312-A Fraccionamiento Gonzalo Guerrero, 97118 Mérida, YUC, Mexico; ^2^Departamento de Ingeniería de Sistemas y Automática, Universitat Politècnica de València, C. Vera 14, 46022 Valencia, Spain

## Abstract

This work proposes a new method for fault diagnosis in electric power systems based on neural modules. With this method the diagnosis is performed by assigning a neural module for each type of component comprising the electric power system, whether it is a transmission line, bus or transformer. The neural modules for buses and transformers comprise two diagnostic levels which take into consideration the logic states of switches and relays, both internal and back-up, with the exception of the neural module for transmission lines which also has a third diagnostic level which takes into account the oscillograms of fault voltages and currents as well as the frequency spectrums of these oscillograms, in order to verify if the transmission line had in fact been subjected to a fault. One important advantage of the diagnostic system proposed is that its implementation does not require the use of a network configurator for the system; it does not depend on the size of the power network nor does it require retraining of the neural modules if the power network increases in size, making its application possible to only one component, a specific area, or the whole context of the power system.

## 1. Introduction

Nowadays, the operators of the control centers of the electrical energy transmission systems are overwhelmed by the great amount of information they are required to analyze to maintain the system in the best operation conditions. When there is an event in the system, the operator, based on the alarms set by the SCADA and the faulty components, tries to make the most accurate diagnosis of the current situation of the system to restore it as soon as possible. That diagnosis can be extremely complicated depending on the number of the faulty components and the operated protection devices. This paper is fundamentally aimed to introduce a method for the implementation of a fault diagnosis system, using artificial neural networks with a modular focus on electrical energy transmission systems, for the purpose of using it as an auxiliary tool for decision making. It is a well-known fact that a fast and accurate diagnosis helps restore the collapsed electrical system quickly.

In the last decades, projects focused on the diagnosis of faults in the power electrical systems, with different neural structures such as Bayesian networks [1], radial basis function networks (RBF) [2, 3], backpropagation networks [4, 5], and SOM networks [6], have been developed with good results but with limitations. One limitation is the enclosed structure of monolithic type, which as the networks increase size implementing a diagnosis of faults becomes more difficult [4, 7, 8].

The fault diagnosis method proposed here consists of two levels of diagnosis for the bus neural modules, the transmission lines, and the transformers and a third level assigned only for the neural module of the transmission lines. The first level of diagnosis checks if the fault occurred in the component undergoing analysis through the correct operation of their own and/or the back-up switches associated to it [9, 10]. The second level of diagnostic verifies whether the fault was in the component under analysis through the correct operation of its own protection schemes or the back-up ones [9, 10]. The result of the two previously established diagnostic levels is used to verify that both levels are true for the fault so it is assigned to the component under analysis; otherwise the fault does not correspond to this component. The third level diagnostic module, assigned only to the neural transmission lines, reinforces the diagnosis of the first two levels of the module by processing the continuous signals and the frequency spectra, the voltage oscillograms, and the current failure of the corresponding line through a neural structure to verify if it really was subject to failure and also to determine the type of the failure (L-g, LL-g, LL, LLL, and LLL-g). A considerable advantage of the proposed diagnostic system is that its implementation does not require the use of a system network configurator. It does not depend on the size of the grid nor on retraining neural modules if the grid increases in size; and it can be applied to a single component, a specific area, or the whole context of the power system. The method is reinforced to issue a more accurate diagnosis since it considers both the analog voltages and the fault currents of the transmission lines, such as the discrete ones (state of switches and relays).

## 2. Description of the Diagnosis Method

**Figure 1:**
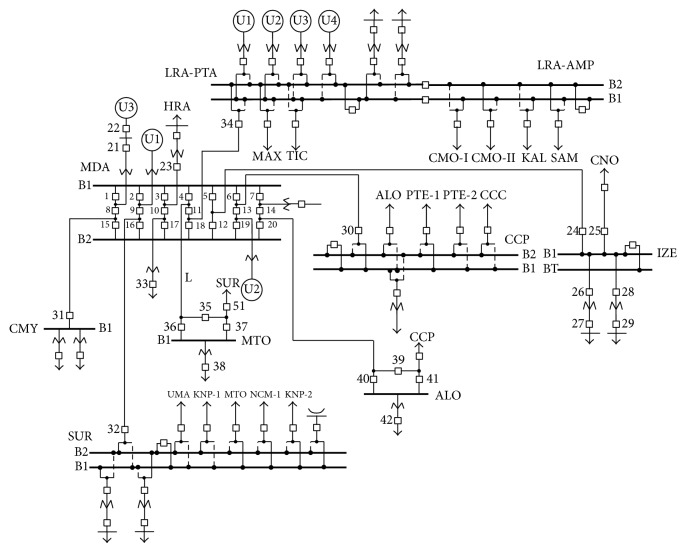
Topology of the interconnection of L.

To explain the proposed method, the Merida “a breaker and a half” substation (MDA-115 kV), which belongs to the Merida Zone of the peninsular area of the Mexican electrical system, is taken as an example. This substation interconnects to the Metropolitan substation (MTO-115 KV) through the L line, as shown in Figure 1. To make it easier, the method will be applied only to the L transmission line [11–13].

### 2.1. First Level of Diagnosis (by Breakers)

The L transmission line referred to is the MDA-73400-MTO, and it is connected at both ends to “a breaker and a half” substations. The primary breakers which connect the L line to both substations MDA-115 and MTO-115, as shown in Figure 1, are MDA INT-4, MDA INT-11, MTO INT-35, and MTO INT-36.

Each component of the electrical network is characterized by a group of protection schemes which protect it against short-circuit fault.

For the L transmission line of the MDA-115 substation, the primary protection scheme is represented by a 21-distance relay for faults between phases and a 21N distance relay for phase-to-ground faults. This kind of protection is typically for radial or long lines; as for grid or short lines, the primary protection can be characterized by an 87L differential relay.

The secondary scheme of protection is implemented by a 67-overcurrent directional relay for faults between phases and a 67N overcurrent directional relay for faults from phase to ground.

The back-up or additional scheme of protection, in this case, is comprised by a 50FI instantaneous overcurrent relay, and it is directly related to each breaker.

Based on the fact that for any activation of a relay the opening of a breaker corresponds if there is a failure in the L transmission line, the knowledge that the neural module will have to learn will be implemented based on the following criterion.

If the failure really happens in L, the primary breakers of both ends INT's MDA-4 and -11 and INT's MTO-35 and -36 should open.

*Sending Side.* If the INT MDA-4 fails, the breakers that should open to avoid the propagation of the fault are MDA-1, 2, 3, 5, 6, and 7.

If the INT MDA-11 fails, the back-up breakers that should open to avoid the propagation of the fault are INT MDA-18 and INT LRA-34.

*Receiving Side.* If the INT MTO-36 fails, the back-up breakers that should open to avoid the propagation of the fault are INT MTO-37 and -38.

If the INT MTO-35 fails, the back-up breakers that should open to avoid the propagation of the fault are INT MTO-37 and INT SUR-51.

#### 2.1.1. Implementation of the Knowledge Base

With the purpose of a better and more adequate handling of the information about the state of the protection breakers and relays, the information taken from the SCADA system is organized creating the data base shown in Table 1. Each component undergoing diagnosis will have a similar data base according to its characteristics.

Based on the database of line L, patterns with the different combinations are created. These patterns characterize the fault in each of the primary and/or the back-up breakers, and they will be used for the training of a backpropagation multilayered neural network [6, 14, 15]. This network will have as input the latter patterns and as output the activation since the fault was in the line under diagnosis, considering only the opening of the breakers on one side (sending side).

This neural structure will be formed by an input layer of 11 neurons and an output layer of only one neuron. It is worth mentioning that this diagnosis is located only at an end of a line (sending side) so it is necessary to locate a similar neural network with the same patterns and the same output at the other end of the line (receiving line).

The combination of the results of the neural networks at both ends (sending and receiving) will issue the final diagnosis of the component, in this case line L. For this combination, a logical decision table is constructed; this table has as inputs the activations of each of the ends of line L and as outputs the verification by breakers that the fault is in the line.

This decision table is implemented by a neural structure formed by an input layer with two neurons and an output layer with a single neuron. A fault will only exist if the activation occurs at one or both ends of the line.

**Figure 2:**
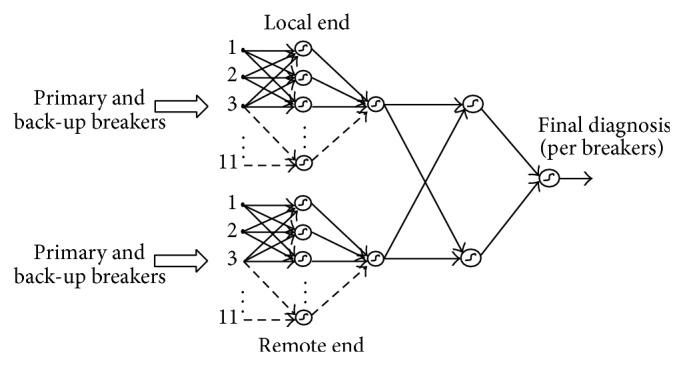
Modular network for the fault diagnosis by breakers in the line.

The complete modular structure for the failure diagnosis, considering only the activation states of each one of the line breakers is shown in Figure 2.

### 2.2. Second Level of Diagnosis (by Relays)

With the above neural network, the existence of a fault in line L is determined only by the opening of the primary and the back-up breakers that are directly related to it. It may be the case that the single state of the breakers cannot determine whether there is actually a fault in this line or not due to the lack of information; as a consequence, it is necessary to reinforce this diagnosis with the validation of the protection schemes directly related to the L line. In this case, there are three protection schemes per primary breaker associated to the line, and remembering that if the failure is really in the L line, at least one relay of a protection scheme at both ends of the line must be activated, except for the 50FI relay associated to the fault of the breaker of the L line and to the activation of at least another relay (21, 21N, 67, and 67N).

#### 2.2.1. Implementation of the Knowledge Base

**Figure 3:**
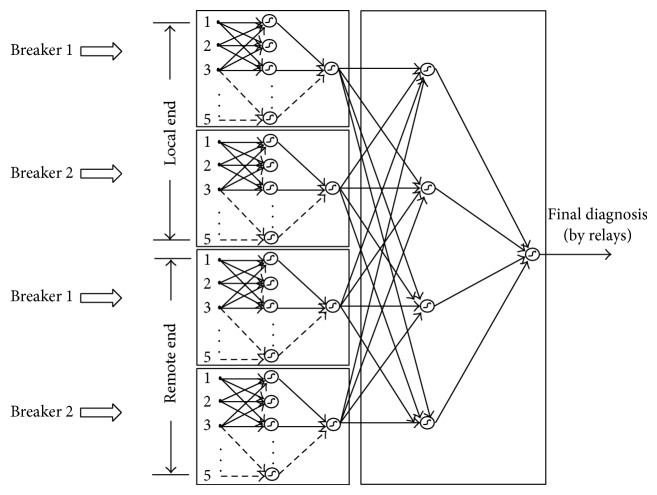
Modular network for the failure diagnosis by protection schemes.

The combination of the activation states of each relay will issue the final diagnosis on each component, in this case line L. Considering the protection schemes, to have a failure in line L one relay, at least, must be activated at both ends. For this combination, a decision logic table is designed; this has as inputs the activation states of each relay and as outputs the final diagnosis for the line, according to the logic state of the protection scheme of each breaker. With the activation of the protection schemes of the line, we can state that the failure occurs in this one. The complete neural structure for the diagnosis, considering exclusively the activation states of the different protection schemes of the line, is shown in Figure 3.

**Figure 4:**
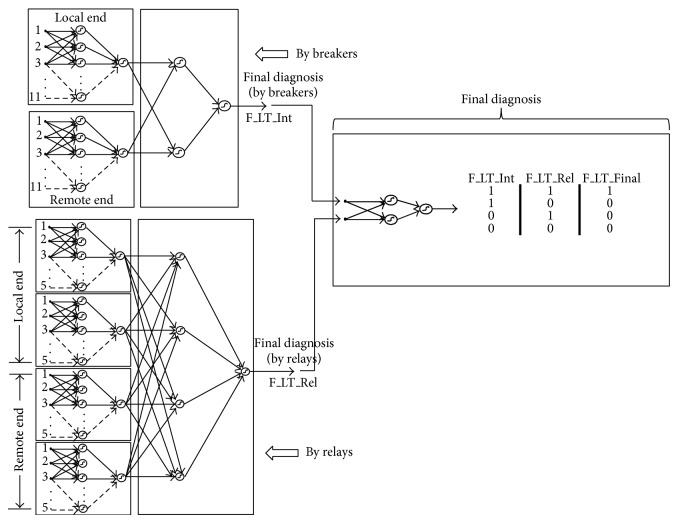
The complete neural structure for the diagnosis connected at both ends with “a breaker and a half” substations.

The logic for failure validation in the line, considering both the breakers and the protection schemes diagnosis, will be implemented by a perceptron neural network, which will have as inputs both breakers and relays diagnosis and as outputs the final diagnosis of line L. To consider a failure in the valid line, it is required that the breaker validation and the protection scheme validation are confirmed; otherwise, the failure in line L will be ruled out. The whole neural structure for the failure diagnosis in lines connected at both ends to “a breaker and a half” substations is shown in Figure 4.

This structure will be implemented by a function on the MatLab software environment, and it will be invoked every time any transmission line suffers any change of state in its own or the backup relays. It is worth mentioning that both for the buses as for the transformers the same implementation procedure described above is used.

## 3. Third Diagnosis Level (for Current Oscillograms and Fault Voltages)

In order to have a more accurate and reliable diagnosis system, the final diagnosis of the faulty transmission lines is reinforced with a third level of verification which processes the voltage oscillograms and the fault currents in the corresponding line [16] as well as the frequency spectra of these oscillograms, through a neural structure, to verify if it really had a fault and at the same time to determine its type (L-g, LL-g, LL, LLL, and LLL-g). This process can be carried out since all the transmission line, suffering a fault, will show fault currents and voltages before it is isolated from the system by its own protection schemes.

**Figure 5:**
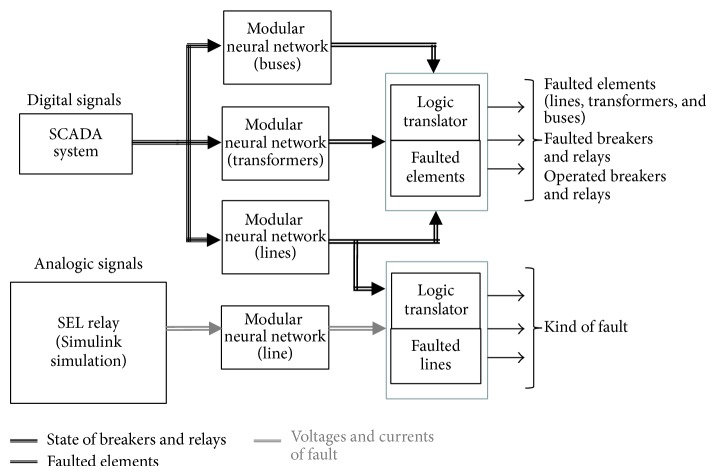
Diagram of the interconnection of the different levels of diagnosis.

The interconnection block diagram for the different diagnosis levels is shown in Figure 5.

### 3.1. Transmission Line Model to Obtain the Types of the Fault

The data base representing the training patterns for the proposed neural structure for the third level of diagnosis will consist of the characterization of each one of the dynamics of the different fault types occurring in a transmission line (L-g, LL-g, LL, LLL, and LLL-g). These dynamics are obtained from simulations carried out in the MatLab PowerSys Blockset [17] with the characteristic parameters of the transmission line being diagnosed, which corresponds to a 13 Km long line operating at a nominal voltage level of 115 kV.

Each one of the types of fault will be characterized by their transient response oscillograms of each phase. The knowledge base will consider the voltage and current oscillograms as training patterns for each one of the fault types that happened whether at the end of the sending line, in the center, or at the end of the receiving line. To explain the procedure, voltage and current oscillograms at the end of the transmission line (at 3 Km) for a phase A to ground type of fault will be simulated so the corresponding graphs can be observed and presented in a data base to train a neural structure which will classify the type of fault occurred in the line and also verify if the fault did happen.

#### 3.1.1. Calculus Methodology

To simulate the voltage and current fault for each one of the different types of faults in the model of the line presented in the PowerSys, a frequency signal of 28.8 KHz will be handled [5, 18]. This signal frequency guarantees a good simulation of the current and voltage signals of analog type which occur in the recorders of events located at the ends of the transmission lines.

The reproduction of signals for simulation purposes, of a 28.8 KHz frequency and a simulation time of 0.1 seconds, corresponds to an integration time of 34.722 *μ*sec and 2880 points for each simulated signal.

The simulation time will be of 0.1 seconds since this time corresponds to 6 cycles of the current or voltage signal where the first two cycles match the dynamic of the signal previous to the fault, the three following cycles correspond to the fault dynamic and the last cycle is where the fault is now released.

#### 3.1.2. Filtering and Sampling Process

It is proven that when handling a 28.8 Khz frequency for the voltage and current signals of the fault, it is possible to reproduce, through simulations, the different types of fault occurring in the transmission line. To condition the voltage and current analog signals, a second-order low-pass filter is included in the model represented in the PowerSys to eliminate high frequencies and so avoid the aliasing problem during the sampling process [19]. To count with the sampled fault voltage and current signals so they represent accurately the real fault voltage and current signals, a decimation of 120 rates is done. This means that a point will be taken as a sample every 120 points of each cycle resulting in 4 points (samples) for each cycle of both voltage and current signals so if the dynamics of the voltage and current faults is characterized by 6 cycles there will be 24 samples which will reproduce accurately the original signals [5].

**Figure 6:**
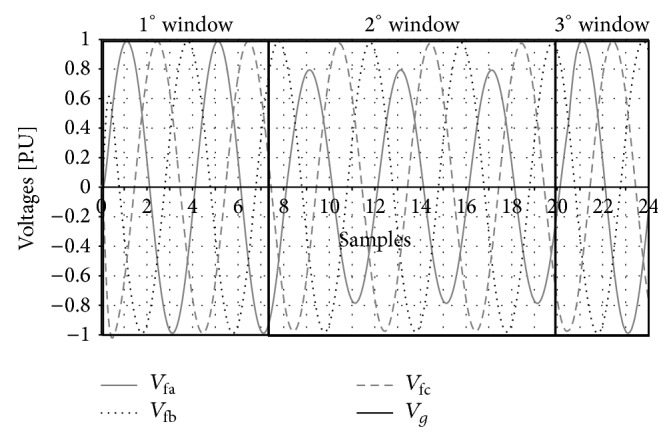
Failure voltages in the different phases and in neutral.

**Figure 7:**
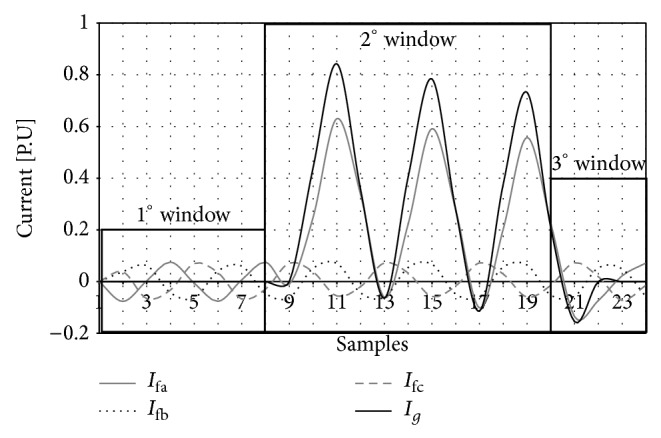
Failure currents in the different phases and in neutral.

The classification of the signals by sectors can be seen in Figures 6 and 7 where the voltage and current dynamics for a phase A to ground fault is represented.

### 3.2. Structure of the Training Data Base (Continual Signals)

To illustrate, it will be shown how to organize the data used as training patterns for the neural structure. The input patterns will be obtained from the simulations corresponding to the type of faults.

The data base will be implemented as follows: for a phase A fault, to ground, the information of the voltage and current of the different phases is represented in Figures 6 and 7, and in this particular case at a distance of 3 Kms from the sending bar of the transmission line mentioned above, it will be grouped as shown in Table 2. The first eight columns represent the voltage and current values of each phase, which, in this specific case, correspond to a phase A to ground fault to a distance of 3 Kms from the sending node of the transmission line. The last four columns represent the type of fault in a binary way, which is referred to, in this case, as a phase A to ground fault.

The structures of the data for phase A to ground fault in the center (6.5 Km) and at the receiving end of the line are placed in a decreasing order. In total, there are 72 training patterns, which characterize a line-to-ground fault, in this case of phase A, in three different places of the line, at the sending end (3 Km), in the middle (6.5 Km), and last at the receiving end of the line (3 Km). Being able to handle three possible positions of the fault in the line provides the neural structure with a generalization capacity. Since with these three possible locations of the fault, it can classify appropriately the type of fault the line is suffering. This structure completes the grouping; it repeats itself for each one of the types of faults; this means there will be 11 groupings of 72 patterns adding up to 792 training patterns. The outputs of each grouping, the same as the first one, represent the type of fault, which they refer to in a binary way.

#### 3.2.1. Neural Structure

The neural structure will be formed by an input layer with 8 inputs, a hidden layer with 14 neurons, and an output layer with 4 neurons. This structure presented the greatest generalization capacity, using the error backpropagation algorithm, for the pattern classification that the neural structure was designed for. Each of the transmission lines in the electric power network will be assigned a similar neural structure which will be trained according to the parameters and characteristics of each line.

### 3.3. Structure of the Training Data Base (Using the FFT)

To design the neural structure considering the frequency spectrum for each one of the different types of faults for the third level diagnosis of the transmission lines, the sampled analog type fault voltage and current signals will be considered, and the frequency spectrum for each one will be obtained using the FFT (fast Fourier transform). Such frequency spectrum will be considered as the input patterns to implement the knowledge base with which the neural structure will be trained.

**Figure 8:**
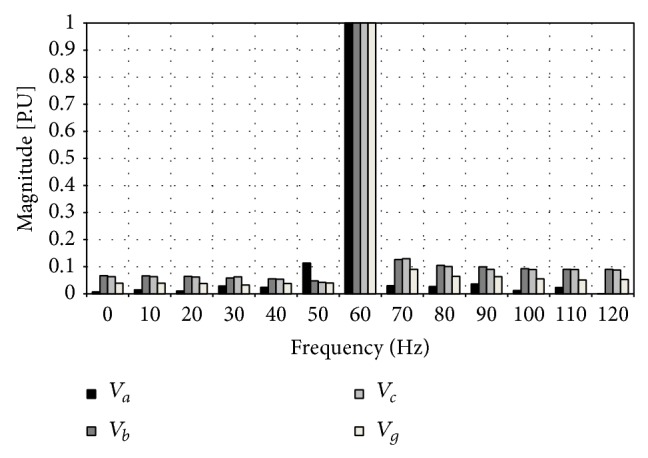
Failure voltages: phase A to ground.

**Figure 9:**
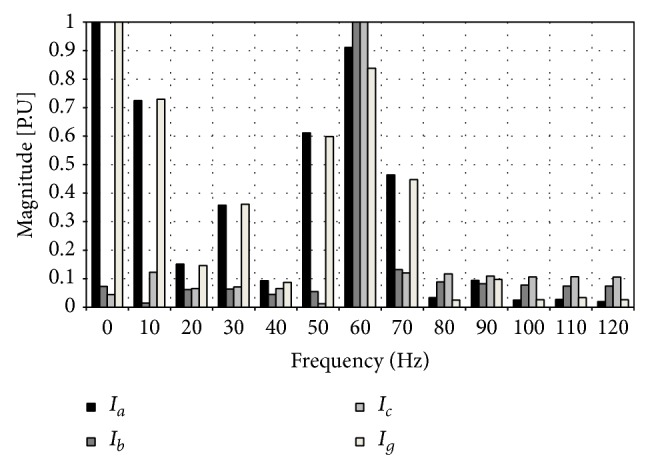
Failure currents: phase A to ground.

The input patterns will be obtained from the frequency spectrum, which correspond to the type of fault. The data base will be represented as follows: for phase A to ground fault the frequency spectrum pertaining to such faults is shown in Figures 8 and 9.

Such spectrum represents fault currents and voltages of phase A to ground at a three-kilometer distance of the sending bar of the transmission line mentioned above. The values of each one of the samples of the spectrum of frequency of the voltage and current fault are ordered the same way as shown in Table 2 for the currents and voltages of the different phases.

The first eight columns show the values of the frequency spectrum of the voltage and current of the fault in each one of the phases where, in this specific case, these values correspond to a phase A to ground fault at a distance of three kilometers from the sending node of the transmission line. The last four columns represent the type of fault in a binary form which refers to, in this case as stated before, a phase A to ground fault.

The spectrum in frequency for phase A to ground fault is placed in a decreasing way in the center (6.5 Km) of the line and at the end of the receiving side of the same line (3 Km). In total, there are 39 training patterns that characterize a fault in the line to ground, in this case of phase A, in three different locations, at the sending end (3 Km), in the middle (6.5 Km), and last at the receiving end (3 Km) of the line. This complete grouping structure repeats itself for each one of the types of fault; this means there are 11 groupings (one per type of fault) of 39 patterns adding up to 429 training patterns. Handling three possible fault locations in the line provides the neural structure with a good generalization capacity since with these three possible fault locations the neural structure is capable of classifying appropriately the type of fault suffered by the line.

The neural structure will be formed by an input layer with 8 inputs, a hidden layer with 14 neurons, and an output layer with 4 neurons. This structure is the same as the one for the analog signals. The difference is that this neural structure will be trained with the spectrum of frequency of the analog signals of the voltages and currents of the fault as inputs. Each of the transmission lines in the electric power network will be assigned a similar neural structure which will be trained according to the parameters and characteristics of each line.

## 4. Implementation of the Diagnostic Method

**Figure 10:**
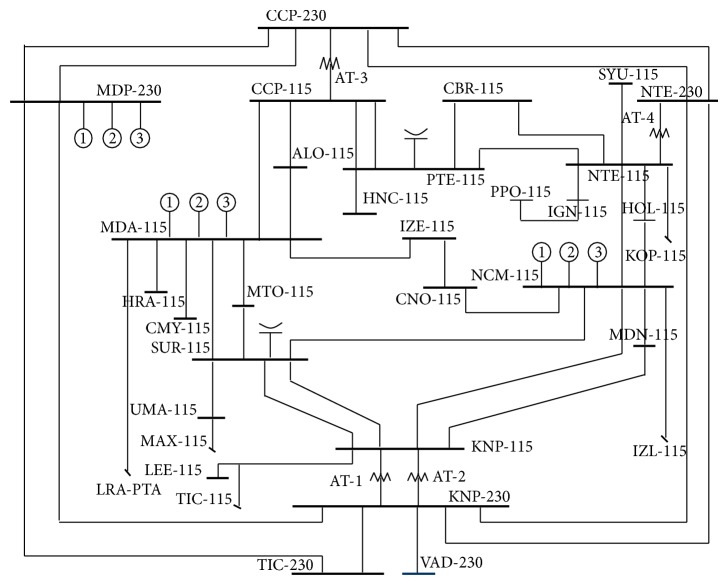
Electric power network for the urban area of the city of Merida, Yucatan, Mexico.

In order to provide a clear representation of the implementation of this fault diagnosis method, Figure 10 presents the unifilar of the electric power network for the urban area of the city of Merida, Yucatan, Mexico, where this method is applied.

**Figure 11:**
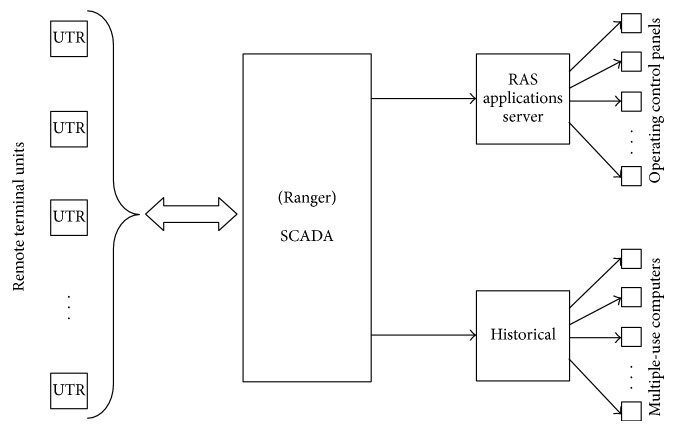
Data acquisition structure of the SCADA.

**Figure 12:**
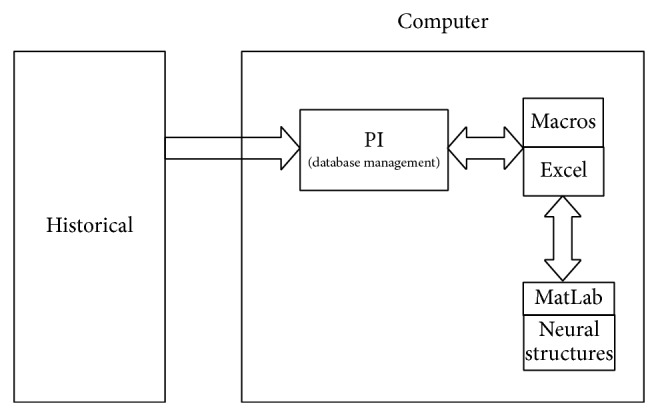
Fault diagnosis system structure.

In accordance with the block diagram showing the interconnection of the different diagnostic levels, presented in Figure 5, the diagnostic method is based on the Excel platform, as this has a complementary application which interacts with the historical database of the SCADA system and which was developed by the supplier of the SCADA software used in the electric power network for the urban area of the city of Merida, Yucatan, Mexico. This platform also interacts with MatLab ambient in order to resolve each of the neural structures corresponding to each element in the electric power network. The structures for acquiring the information from the historical database of the SCADA, and the fault diagnosis system, are shown in Figures 11 and 12.

Table 3 shows the list of faults detected by the diagnostic system, from January to June of the current year, for the different components of the electric power network (buses, transformers, and lines).

## 5. Resulting Information Produced by the Fault Diagnosis System

The following are some of the faults reported by the fault diagnosis system from January to June of 2014, which include simultaneous faults, breaker faults, and relay faults.

### 5.1. Simultaneous Faults

Fault in bus-2 of the PTE substation, with simultaneous tripping of the PTE-73340-CBR, PTE -73590-CCP lines and the PTE-T2 transformer (see Boxes 1, 2, and 3).

### 5.2. Failure of Primary Protections

Fault in LT NTE-73430-IGN of the NTE substation, with failure of primary protections (see Box 4).

### 5.3. Failure of Primary Breakers

Fault in LT IGN-73490-PPO of the San Ignacio substation, with failure of primary switch (see Box 5).

To prove the efficiency of the proposed method, the following example is solved through the global neural structure (monolithic type) formed by three layers. The first layer has 43 neurons corresponding to the breakers, 10 neurons corresponding to the buses, 7 neurons corresponding to the transmission lines, and 69 neurons corresponding to the relays of the components which will be diagnosed adding in total 129 neurons in the input layer.

The hidden layer is formed by 250 neurons and the output layer by 17 neurons each one related to the component to be diagnosed. The neural structure is trained by the patterns that represent the normal operation of the network, the operation with the simple fault of the breaker and with the simple fault of the relays. Under these training patterns the network responds adequately both for simple faults as for breakers and relays. For double faults (two breakers and two relays or one breaker and one relay) the neural structure does not respond adequately so it is necessary to train it with double fault patterns. For triple or more faults, it is necessary to introduce patterns with these kinds of faults to the neural structure so it can show generalization capacity with these faults.

From the above, it can be noticed that if the size of the network increases so will the neural structure, so for complex systems this type of global structure will become more complex. The advantage of the proposed method is that it only has three neural structures: one for the buses, one for the lines, and one for the transformers.

Another advantage of this method is that if the network increases its size, retraining the neural structures is not necessary. One additional advantage is that to reinforce the diagnosis of the transmission lines, both the discrete (breakers and relays states) signals and the analog ones (fault voltage and current faults) are considered.

Also to compare the proposed method to logical reasoning methods, the above example is solved using 17 hypotheses, one per each component capable of failure. Each hypothesis is formed by three basic rules with their corresponding conditions for normal operation of the flawed component, breakers fault, and relays fault. The result obtained with this method equals the one obtained with the proposed method with the difference that the proposed method only uses three neural modules, one per each type of component versus the 17 hypotheses generated from the logical reasoning method. For the logical reasoning method if the amount of system components grows, a new hypothesis per component must be generated while with the proposed method there are still only three neural modules. So the proposed method unlike the logical reasoning method uses the analog signals and the frequency spectrum of the voltage and current faults corresponding to the transmission lines to confirm their faults if there is no enough information of the breakers and relays coming from SCADA.

## 6. Conclusion

Applying this new method gives a diagnosis at component level since there are three generic modules that will be called depending on the type of component to be diagnosed. This allows a diagnosis per component, per zone, or for the whole context of the electrical system. The method is reinforced considering the diagnosis of the corresponding transmission line, both the oscillograms and the frequency spectrum of the voltage, and current of the fault, through a neural structure, to verify if a fault really happened and at the same time to determine the type of the fault (L-g, LL-g, LL, LLL, and LLL-g). This is possible since each one of the generic modules will be called every time the primary and/or the back-up relays of each one of the components (lines, transformers, and buses) change state. It is also observed that it is plausible to use this neural modular structure which can be used as a tool for the operators of the control centers.

## Figures and Tables

**Box 1:**
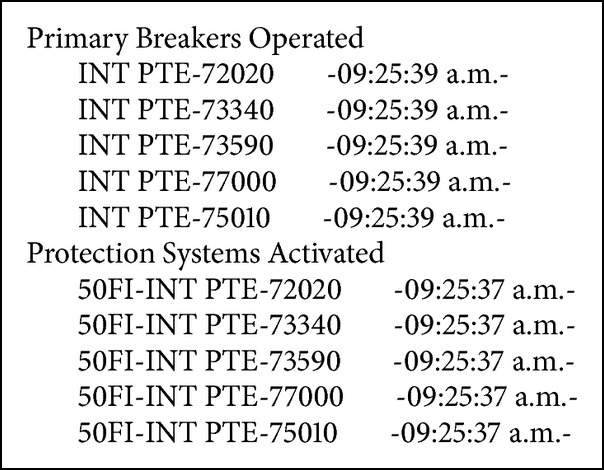
Fault in MDA BUS-1.

**Box 2:**
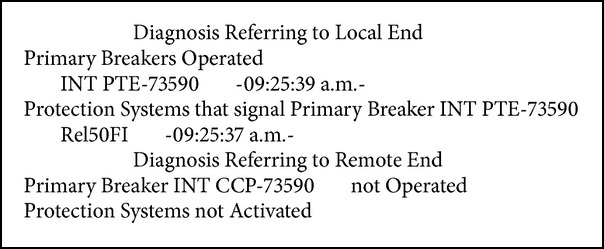
Fault in LT PTE-73590-CCP.

**Box 3:**
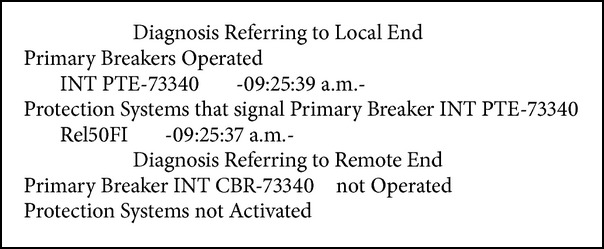
Fault in LT PTE-73340-CBR.

**Box 4:**
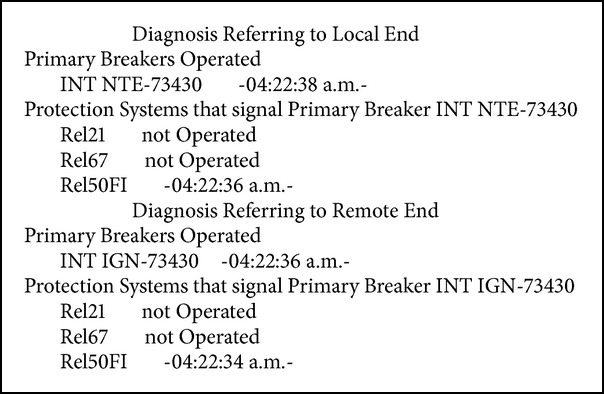
Fault in LT NTE-73430-IGN.

**Box 5:**
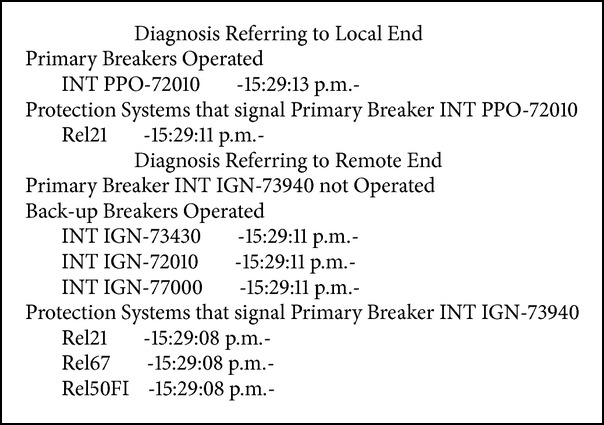
Fault in LT PPO-73490-IGN.

**Table 1:** Data base for the L neural module.

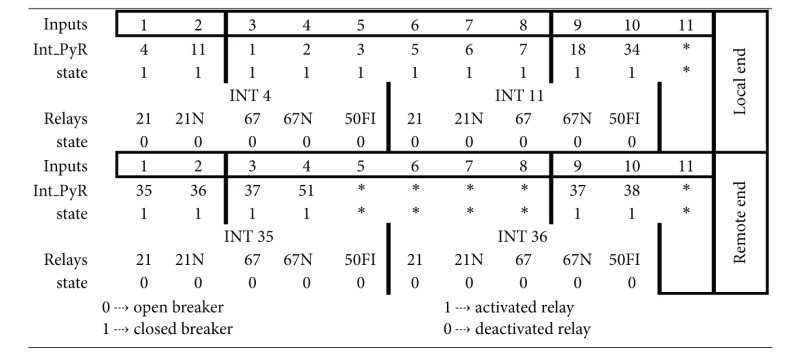	

**Table 2:** Training data base structure.

	1	2	3	4	5	6	7	8	Output				Samples
	*V*_fa_	*I*_fa_	*V*_fb_	*I*_fb_	*V*_fc_	*I*_fc_	*V*_*g*_	*I*_*g*_	*F*_fa_	*F*_fb_	*F*_fc_	*F*_*t*_	
Fa_n													
1° window	−0.000641867	−3.89083*E − *05	−0.001196415	−0.000115795	0.001838282	0.000154703	−1.28535*E − *16	2.94942*E − *19	0	0	0	0	**1**
	0.973501524	−0.076403259	−0.341666999	0.038533053	−0.631834526	0.037870206	−6.91105*E − *14	1.37198*E − *15	0	0	0	0	**2**
	0.156566273	0.002870909	−0.922627797	0.062640947	0.766061524	−0.065511856	3.39279*E − *14	8.94746*E − *17	0	0	0	0	**3**
	−0.977512891	0.075485554	0.364054716	−0.043150787	0.613458175	−0.032334767	6.83136*E − *14	−1.3745*E − *15	0	0	0	0	**4**
	−0.130876088	−0.005686783	0.913497316	−0.064549946	−0.782621228	0.070236728	−3.57852*E − *14	−5.31128*E − *17	0	0	0	0	**5**
	0.980884519	−0.076176694	−0.388121653	0.041806383	−0.592762866	0.034370311	−6.73465*E − *14	1.37569*E − *15	0	0	0	0	**6**
	0.105208833	0.006836513	−0.903372829	0.06041348	0.798163996	−0.067249992	3.75514*E − *14	1.70883*E − *17	0	0	0	0	**7**
	−0.983615903	0.075157898	0.4118215	−0.046419404	0.571794403	−0.028738494	6.63519*E − *14	−1.37607*E − *15	0	0	0	0	**8**

2° window	−0.085390516	−0.009294317	0.893416538	−0.062237336	−0.812232671	0.071861669	−3.93077*E − *14	0.000694649	1	0	0	1	**9**
	0.794482606	0.244448121	−0.41313357	0.051890314	−0.528477208	0.037597989	−6.87857*E − *14	0.435579785	1	0	0	1	**10**
	0.07857321	0.608565667	−0.883929869	0.070899477	0.824708251	−0.055939163	4.14844*E − *14	0.812910497	1	0	0	1	**11**
	−0.780159859	0.298755448	0.434207722	−0.044746113	0.504871283	−0.020256067	6.79103*E − *14	0.304993572	1	0	0	1	**12**
	−0.043255913	−0.097347222	0.870970136	−0.061615551	−0.839513012	0.071455966	−4.29452*E − *14	−0.113640457	1	0	0	1	**13**
	0.795320502	0.185590131	−0.458668486	0.053684383	−0.484590465	0.032579109	−6.65731*E − *14	0.354537359	1	0	0	1	**14**
	0.035436192	0.539746239	−0.860491626	0.066775976	0.850674399	−0.058884817	4.49033*E − *14	0.713893895	1	0	0	1	**15**
	−0.784244924	0.2154572	0.479820124	−0.049547695	0.460982222	−0.018303638	6.56191*E − *14	0.192631481	1	0	0	1	**16**
	−0.003700022	−0.159990825	0.846601051	−0.060247995	−0.864070832	0.071439863	−4.64465*E − *14	−0.193629071	1	0	0	1	**17**
	0.794671736	0.146470088	−0.503122432	0.055762758	−0.439532497	0.027911125	−6.42076*E − *14	0.300094087	1	0	0	1	**18**
	−0.007413499	0.488236623	−0.834598405	0.062873078	0.874412279	−0.061256088	4.82739*E − *14	0.638497717	1	0	0	1	**19**
	−0.785563286	0.149009345	0.523946543	−0.053838314	0.415652817	−0.015916624	6.31095*E − *14	0.103483911	1	0	0	1	**20**

3° window	0.036283926	−0.206004065	0.819911852	−0.05837136	−0.886263953	0.071582108	−4.979*E − *14	−0.251041408	0	0	0	0	**21**
	0.989358754	−0.071865898	−0.570113161	0.053793186	−0.417184944	0.019281513	−5.86319*E − *14	0.002047061	0	0	0	0	**22**
	−0.100636605	0.022449661	−0.801635165	0.04975652	0.900541685	−0.072491386	5.0136*E − *14	1.41371*E − *06	0	0	0	0	**23**
	−0.982484998	0.071828401	0.589385182	−0.058290945	0.39193272	−0.013785476	5.68487*E − *14	−2.15631*E − *07	0	0	0	0	**24**

	⋮		⋮		⋮		⋮		⋮			⋮	
							
							

**Table 3:** List of faults detected by the diagnostic system January–June 2014.

Year 2014		Faulty element	Response of fault diagnosis system
January	Lines		
	Buses		
	Transformers	NCM T5	Exact diagnosis

February	Lines	NTE 73430 IGN	Exact diagnosis
		IGN 73490 PPO	Exact diagnosis
	Buses		
	Transformers	NTE AT4	Exact diagnosis

March	Lines	KNP 73880 MDN	Exact diagnosis
	Buses		
	Transformers	NTE T3	Exact diagnosis
		KOP T3	Exact diagnosis

April	Lines	TIC 73070 LRA-PTA	Exact diagnosis
		PTE 73590 CBR	Exact diagnosis
		PTE 73330 NTE	Exact diagnosis
	Buses	PTE BUS-2	Exact diagnosis
	Transformers	IGN T1	Exact diagnosis

May	Lines		
	Buses		
	Transformers	NCM T6	Exact diagnosis

June	Lines	NCM 73150 CON	Exact diagnosis
		CNO 73520 IZE	Exact diagnosis
	Buses	CNO BUS-1	Exact diagnosis
	Transformers	CNO T1	Exact diagnosis
		CNO T2	Exact diagnosis

## Abbreviations and Acronyms

L-g: Phase-to-ground fault
LL-g: Phase-to-phase-to-ground fault
LL: Phase-to-phase fault
LLL: Three-phase fault
LLL-g: Three-phase-to-ground fault
L: Transmission line under diagnosis
INT: Breaker
Int PyR: Primary and back-up breakers
YInt_e: Fault in line by breakers, local end
YInt_r: Fault in line by breakers, remote end
Yrel_e: Fault in line by relays, local end
Yrel_r: Fault in line by relays, remote end
F LT Int: Fault in line by breakers
F LT Rel: Fault in line by relays
$I_{\text{fa}}$: Fault current in phase A to ground
$I_{\text{fb}}$: Fault current in phase B to ground
$I_{\text{fc}}$: Fault current in phase C to ground
$I_{g}$: Fault current to ground
$V_{\text{fa}}$: Fault voltage in phase A to ground
$V_{\text{fb}}$: Fault voltage in phase B to ground
$V_{\text{fc}}$: Fault voltage in phase C to ground
$V_{g}$: Fault voltage to ground
$V_{a}$: Fault voltage spectrum phase A to ground
$V_{b}$: Fault voltage spectrum phase B to ground
$V_{c}$: Fault voltage spectrum phase C to ground
$V_{g}$: Fault voltage spectrum to ground
$I_{a}$: Fault current spectrum phase A to ground
$I_{b}$: Fault current spectrum phase B to ground
$I_{c}$: Fault current spectrum phase C to ground
$I_{d}$: Fault current spectrum to ground.
